# Laetoli Footprints Preserve Earliest Direct Evidence of Human-Like Bipedal Biomechanics

**DOI:** 10.1371/journal.pone.0009769

**Published:** 2010-03-22

**Authors:** David A. Raichlen, Adam D. Gordon, William E. H. Harcourt-Smith, Adam D. Foster, Wm. Randall Haas

**Affiliations:** 1 School of Anthropology, University of Arizona, Tucson, Arizona, United States of America; 2 Department of Anthropology, University at Albany–SUNY, Albany, New York, United States of America; 3 Department of Anthropology, Lehman College, Bronx, New York, United States of America; 4 Division of Vertebrate Paleontology, American Museum of Natural History, New York, New York, United States of America; University of Delaware, United States of America

## Abstract

**Background:**

Debates over the evolution of hominin bipedalism, a defining human characteristic, revolve around whether early bipeds walked more like humans, with energetically efficient extended hind limbs, or more like apes with flexed hind limbs. The 3.6 million year old hominin footprints at Laetoli, Tanzania represent the earliest *direct* evidence of hominin bipedalism. Determining the kinematics of Laetoli hominins will allow us to understand whether selection acted to decrease energy costs of bipedalism by 3.6 Ma.

**Methodology/Principal Findings:**

Using an experimental design, we show that the Laetoli hominins walked with weight transfer most similar to the economical extended limb bipedalism of humans. Humans walked through a sand trackway using both extended limb bipedalism, and more flexed limb bipedalism. Footprint morphology from extended limb trials matches weight distribution patterns found in the Laetoli footprints.

**Conclusions:**

These results provide us with the earliest direct evidence of kinematically human-like bipedalism currently known, and show that extended limb bipedalism evolved long before the appearance of the genus *Homo*. Since extended-limb bipedalism is more energetically economical than ape-like bipedalism, energy expenditure was likely an important selection pressure on hominin bipeds by 3.6 Ma.

## Introduction

Ever since Darwin [1], bipedal walking has been considered the defining feature of the human lineage. However, how and why this unique form of locomotion evolved remains the subject of considerable debate. In particular, debates over the origins and evolution of bipedalism revolve around whether early bipeds walked with energetically economical human-like extended limb biomechanics, or with more costly ape-like bent-knee, bent-hip (BKBH) kinematics [2]. If early hominins used a BKBH gait, then we must account for the persistence of an energetically costly form of bipedal walking until the evolution of the genus *Homo*. The Laetoli footprints may help resolve this debate, since they record the footsteps of at least two, and possibly three individuals who walked bipedally across wet ashfall approximately 3.6 million years ago [3], [4]. These prints represent the earliest *direct* evidence of bipedalism in the fossil record, yet no study to date has demonstrated exactly how these hominins walked.

For decades, researchers have argued over whether the Laetoli hominins walked with a modern human-like extended limb gait [3], [5]–[10], or a more ape-like form of bipedalism [11]–[14]. Most recently, the discovery of 1.5 Ma footprints from Kenya provides new evidence that the Laetoli prints were not completely modern in weight transfer and morphology [15]. If true, then an energetically costly form of bipedalism evolved and persisted in early hominins until the evolution of the genus *Homo* [16]. Alternatively, if the Laetoli prints were made by extended-limb bipeds, then, by 3.6 Ma, selection acted to reduce energy costs of locomotion in hominins.

Most previous studies of the Laetoli prints, however, were qualitative and did not test specific biomechanical hypotheses about early hominin gait. Kullmer et al. [17] performed a quantitative comparison of one Laetoli footprint (G1-36) with three footprints made by a single human subject walking with different postures. Their results suggest that Laetoli hominins did not walk like modern humans, however their sample size was very small (one human print for each posture studied) and there is not enough kinematic information to generalize their results to the Laetoli trackways. More recent quantitative work using geometric morphometrics techniques [15], [18] may conflate differences in foot anatomy and shape with differences in locomotor biomechanics. Without using experimental analyses, it is difficult to determine which landmark differences between Laetoli and more recent fossil footprints are due simply to differences in foot morphology, and which are, in fact, due to differences in kinematics. Thus, a thorough experimental-based analysis of the Laetoli prints may resolve the debate over hominin biomechanics, and therefore help clarify the importance of selection for reduced energy costs of locomotion prior to the evolution of the genus *Homo*.

Here, we present the results of the first experimental analysis of footprints in a sample of humans walking with different gaits and compare our results to the Laetoli prints. Eight human subjects (mean body mass [SD] = 65.6 [6.1] kg) walked through a 5 m trackway filled with 15 cm of fine grained sand (0.075 mm–0.70 mm diameter). Although our subjects are likely heavier than the earlier hominins that generated the Laetoli prints, body mass does not appear to have an effect on footprint morphology [19]. We compared footprints made by subjects walking with a normal, extended limb gait, and with a bent-knee, bent-hip (BKBH) ape-like gait at their preferred speeds with sand water content of 6–8% (see supplementary materials Text S1 for hind limb kinematic data from these experiments). These substrate conditions match those of Laetoli, which are described as similar to damp, fine to medium grained sand [20]. We also examined the effects of increased speed and increased substrate moisture (10–12% water) on footprint morphology. We tested the hypothesis that a BKBH gait alters body weight transfer and produces significantly different footprint morphology than an extended limb gait. We predicted that Laetoli footprint morphology would match humans walking with one of these gaits. To test this prediction, we scanned each human print using a 3D laser scanner (Microsribe® MLX 6DOF digitizer with attached Microscan laser sensor system), and compared the maximum depths of the fore- (toe) and aft- (heel) sections of the prints. These values were calculated after leveling each print, since prints were made over a substrate that had a very small grade (mean grade = 1.49%±0.16%). We compared our experimental data with Laetoli footprint depths calculated from contour maps of the footprint trails [21] (Table S1). Additionally, we captured kinematic and kinetic data to determine how walking biomechanics influence footprint morphology (see supplementary materials Text S1).

## Results and Discussion

**Figure 1:**
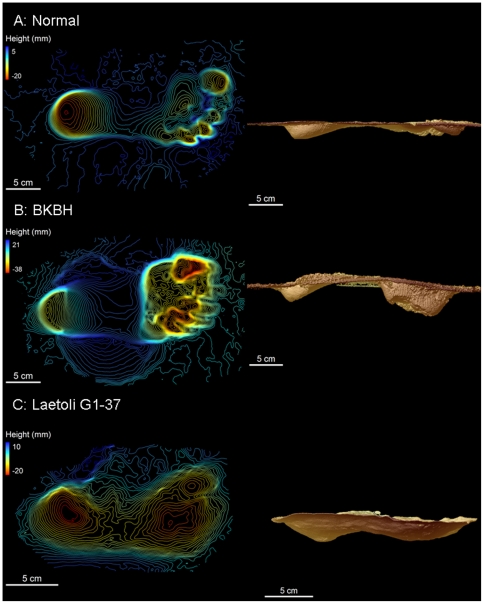
Three dimensional scans of experimental footprints and a Laetoli footprint. Contours are 1 mm. A) Contour map of modern human footprint (Subject 6) walking with a normal, extended limb gait and side view of normal, extended limb footprint. B) Contour map of modern human footprint (Subject 6) walking with a BKBH gait and side view of BKBH print. C) Contour map of Laetoli footprint (G1-37) and side view of Laetoli footprint (G1-37). Note the difference in heel and toe depths between modern humans walking with extended and BKBH gaits. Laetoli has similar toe relative to heel depths as the modern human extended limb print.

**Figure 2:**
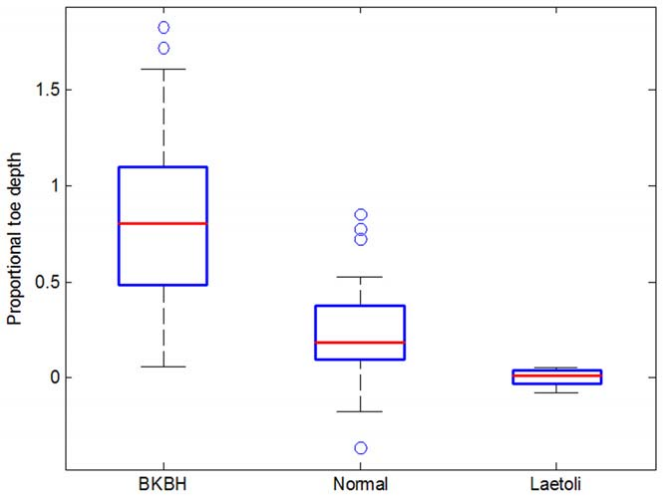
Toe depths as a fraction of heel depth for BKBH, normal walking and Laetoli prints. Laetoli values were calculated using values from topographic maps. Note that the values for Laetoli fall within the range of human normal prints but outside of the range of human BKBH prints.

There is a significant difference in the relative depth of the toes in BKBH compared to extended limb prints (Figs. 1 and 2; Table 1). BKBH footprints from humans walking at preferred speeds have toe depressions that are 76.69%±8.35% lower than the heel (calculated as toe depth as a percentage of heel depth; Table 1). When walking with an extended hind limb at preferred speeds, toe depressions are, on average, 22.36%±4.28% below the heel. Thus, BKBH gaits generate significantly greater toe relative to heel depths compared to extended limb gaits. Speed influences print morphology, with faster speeds leading to deeper toe depressions in both gaits, however between-gait differences are not significant (Table 1). Substrate moisture content also alters footprint morphology (Table 1). Wetter substrates lead to greater toe depths, regardless of gait, however, BKBH gaits have toe depths that are significantly greater than extended limb postures (Table 1).

**Table 1:** Proportional toe depths of human footprints.

Side	Speed	Moisture	Normal	SEM	BKBH	SEM	p-value
Right	p	d	0.27	0.04	0.77	0.12	<0.001
Left	p	d	0.19	0.06	0.76	0.12	<0.001
Both	p	w	0.48	0.11	0.81	0.12	<0.05
Right	f	d	0.98	0.33	0.51	0.12	0.12
Left	f	d	0.38	0.08	0.67	0.16	0.07

p is preferred walk; f is fast walk; d is dry (6–8% water); w is wet (10–12% water).

Values are toe depths as a fraction of heel depth. P-values are for T-Tests comparing BKBH and extended (normal) datasets.

**Figure 3:**
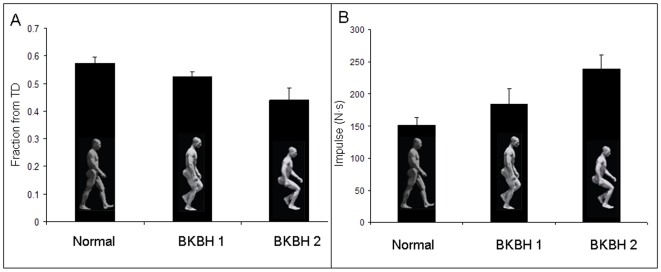
Center of pressure (COP) position and ground reaction force impulse in humans walking with different limb postures. A) COP movement during bipedal walking. Fraction from touchdown (TD) is the mean time as a fraction of stance phase when the COP passes anterior to the metatarsal heads (error bars are SEMs). B) Late stance impulses during bipedal walking. Values are mean for the ground reaction force impulse *after* the COP passes anterior to the metatarsal heads (error bars are SEMs). Normal is extended limb walking, BKBH 1 is walking with a slightly flexed knee and hip, BKBH 2 is walking with a deeply flexed knee and hip.

The difference in print morphology is related to a fundamental difference in body weight transfer between extended limb and BKBH gaits. The center of pressure (COP) is the point of ground reaction force application under the foot. As the COP travels from the heel to the toe during stance phase, its path, and the magnitude of ground force at any given moment, determines the amount of substrate displacement at a given position under the foot. During walking, as the COP shifts anterior to the metatarsal heads, the metatarso-phalangeal joint flexes, and because of the stiff longitudinal arch, the entire foot posterior to the metatarsal heads lifts off the ground [22]. In a BKBH gait, the COP passes the metatarsal heads significantly earlier in the step (i.e., closer to heel-strike; Paired t-test p<0.001), and the ground reaction force impulse after the COP passes the metatarsal heads is significantly greater compared to extended limb gaits (Fig. 3; Paired t-test p<0.001). The larger impulse when the body is supported by the forefoot explains the increased toe-depth in BKBH footprints. Since most researchers agree that the Laetoli prints exhibit evidence of a longitudinal arch [5], [6], [8], [10], [23]–[26] (but see [13]–[15] and supplementary materials Text S1; Figs. S1, S2), an analysis of relative toe depths should provide unequivocal evidence of limb posture in these early hominins.

The Laetoli prints have toe depths that are generally shallower than heel depths, however, the trackways were made on a very slight grade (3.12%±0.94%; see supplementary materials Text S1). After adjusting for this grade with the same procedures used for the experimental footprints (see supplementary materials Text S1 and above), mean toe depths for the G1 set of prints are generally equal to mean heel depths (0.11%±1.61% shallower than heels [0.00 mm±0.02 mm]), resembling weight transfer in a modern human-like extended limb gait more than a BKBH gait (Figs. 1 and 2; Supporting Table S1). In fact, while Laetoli proportional toe depths fall within the range of normal human patterns, Laetoli data fall outside of the range of human proportional toe depths made during BKBH trials (Fig. 2). We did not analyze the G2/3 set of footprints, since they were likely made by two individuals walking one behind the other, rendering their morphology less suitable for biomechanical determinations [9], [11], [27]. There are two other caveats that must be considered in this analysis. First, bioturbation is evident in some of the Laetoli G1 prints [23], and could impact our results. However, much of the bioturbation occurred on the rims of the prints and does not greatly impact the internal morphology of most prints [23], [24]. Second, the thickness of the substrate at Laetoli varies along the print trail [28], which may have an effect on footprint depths [19]. However, the similarity of proportional depths across the G1 trail (Table S1) suggests that differences in substrate thickness did not impact our analysis. Finally, the Laetoli prints offer no evidence that these individuals were walking at fast speeds, since high speed walking also produces prints with much larger toe compared to heel depths for both gaits (see Table 1). Therefore, we conclude that the Laetoli hominins walked with an extended limb gait at speeds consistent with previous predictions (i.e., preferred or slow speeds) [8]–[10], [26], [29]–[32]. The relative toe depths of the Laetoli prints show that, by 3.6 Ma, fully extended limb bipedal gait had evolved. Thus, our results provide the earliest unequivocal evidence of human-like bipedalism in the fossil record.

Hypotheses for the origins of bipedalism often focus on selection for energy economy in early hominins [16], [33]. Energetic hypotheses are based on the reduced locomotor costs of humans compared to apes walking with BKBH gaits, and therefore, compared to ape-like pre-hominin ancestors [16]. Human walking is inexpensive primarily because extended hind limb joints reduce external moments acting at the joints, and therefore, reduce the amount of muscle force required to support body weight [16], [34]. In addition, extended-limb walking reduces joint reaction forces [35] and reduces total body heat loads compared to BKBH walking [36]. By 3.6 Ma, hominins at Laetoli, Tanzania walked with modern human-like hind limb biomechanics, suggesting that selection for energetically economical bipedalism occurred prior to the evolution of the genus *Homo*. It is likely that reduced energy costs associated with extended limb bipedalism allowed early hominins to increase ranging distances during times of forest fragmentation [37] without enduring greatly increased energy costs.

While our results show that Laetoli hominins walked with human-like kinematics, we still cannot be sure of which hominin taxon made the footprints. Many researchers suggest that *Australopithecus afarensis* made the footprint trails [6], [7], [11], although this hypothesis is disputed by others based on differences between print morphology and fossilized foot remains [10], [38]. If *Au. afarensis* did make the Laetoli footprints, then our results support the hypothesis that this species walked with relatively human-like hip and knee extension [39], [40], and that kinematically human-like bipedalism is compatible with adaptations for arboreality found throughout the australopith skeleton [2]. Thus, settling the dispute over the taxonomic identification of the makers of the Laetoli prints will clarify debates surrounding fossil hominin post-cranial material and locomotor behavior [11], [39].

Finally, although our results clearly demonstrate that human-like bipedal kinematics had evolved by 3.6 Ma, the Laetoli prints cannot provide detailed information regarding the locomotor mechanics of earlier hominins. The recently published skeletal material attributed to *Ardipithecus ramidus* raises the hypothesis that, prior to 4.4 Ma, at least one lineage of hominins walked with kinematics that differed greatly from those of modern humans and other later hominins [41]. However, until further functional morphological analyses are performed, it is difficult to assess the likely biomechanics of *Ar. ramidus*. Thus, based on the results of this study, kinematically human-like bipedalism clearly evolved within the first three to four million years of hominin evolution. However, we are left with two possible scenarios for the origins and evolution of bipedalism. First, if a detailed functional analysis supports the hypothesis that *Ar. ramidus* was a habitual bipedal hominin that walked with flexed lower limbs, then energetic selection pressures likely became strongest after the origins of bipedalism, but prior to 3.6 Ma. This finding would not necessarily rule out the energy hypothesis for the origins of bipedalism [34], but would suggest that early bipeds were less energetically economical than modern humans. Second, if *Ar. ramidus* was not a bipedal hominin, then the earliest bipeds may have walked with extended limb joints, suggesting that early bipedalism was as energetically economical as that of later hominins, including those that made the Laetoli footprints. Testing these hypotheses requires more detailed fossil evidence from the earliest hominins. However, our analysis of the Laetoli prints refines the timing of the evolution of human-like bipedal mechanics in the fossil record. Future analyses of fossil remains, and future fossil discoveries, will no doubt improve our understanding of just how early human-like bipedal mechanics evolved, and therefore, help us determine the importance of selection for energy economy during the early evolution of bipedal walking.

## Methods

Eight subjects participated in this study (see Table S2). All methods were approved by the University of Arizona Human Subjects Committee and subjects gave their written informed consent prior to participation.

All subjects were asked to walk at their preferred walking speed through a sand trackway (see Table S3). After making two passes down the walkway at their preferred speed, subjects were asked to walk with a bent-knee, bent-hip (BKBH) gait at their preferred speed. Subjects also repeated the BKBH trial twice. The trackway was 15 cm deep and 5 m long. It was filled with mostly fine sand, with coarser grained sand confined to the beginning and end of the trackway. Grain sizes ranged from <0.074 mm to 0.7 mm in diameter. After walking at their preferred speeds, a subset of the sample was asked to walk at a fast speed using both gaits. A different subset of the sample was asked to walk in both gaits at their preferred speeds after the moisture content of the sand was increased by ∼4%. Moisture content of sand was measured using a HydroSense (Campbell Scientific CD 620) soil moisture system. For low moisture trials, moisture content was 6–8%. For high moisture trials, moisture content was 10–12%. To determine moisture content, the moisture probe was inserted into the footprint after each scan was taken.

Prior to trackway trials, reflective markers were affixed to the hip, knee, ankle, heel, hallux, 1^st^ metatarsal head, and shoulder. Subjects were filmed walking through the trackway using a six-camera Vicon motion analysis system (200 Hz). Maximum, minimum, and average joint angles were calculated for the hip and knee during all trials (Table S4).

After each pass through the trackway, footprints were digitized using a 3-dimensional laser scanner (Microscribe MLX 6DOF digitizer with attached Micorscan laser sensor system). At least two prints (left and right) for each trial were scanned. For each scanned print, four points were selected from the sand around the print: two points medial and lateral to the toes, and two points medial and lateral to the heel. A plane representing the sand substrate was generated based on those four points using a least-squares fit in Microscan Tools. The 3D coordinates of the print and plane were then imported into R where the plane and print were reoriented such that the plane was level. Finally, the depths of the deepest point in the heel and in the toe were extracted from the leveled print, and proportional toe depth was calculated as a fraction of the depth of the heel.

A second sample of subjects (n = 6) were recruited to determine the kinetics of BKBH walking. Subjects walked over a forceplate (AMTI; 4000 Hz) and were simultaneously filmed with a six-camera Vicon motion analysis system (200 Hz). Prior to walking trials, reflective markers were affixed to the hip, knee, ankle, heel, hallux, 1^st^ metatarsal head and shoulder. Subjects walked at preferred speeds using normal extended limb bipedalism as well as two BKBH trials (one with light and one with deep knee and hip flexion; Table S5).

Center of pressure data were collected from force plate recordings for each trial. Combing kinematic and kinetic data, we calculated the time of heel strike (touchdown) and the time (relative to heel strike) that the COP passed anterior to the metatarsal heads. We also calculated the total impulse of the ground reaction force from the time the COP passed the metatarsal heads to toe-off. These values provide a biomechanical measure of how toe depths are generated. For more information on methods see supplementary materials Text S1.

## Supporting Information

Text S1 Supporting Text
(0.02 MB PDF)

Table S1 Laetoli footprint data
(0.03 MB DOC)

Table S2 Subject information
(0.03 MB DOC)

Table S3 Sample size information for footprint analyses
(0.03 MB DOC)

Table S4 Speed and joint angles for trackway trials. Values are means (SEM) for all subjects.
(0.03 MB DOC)

Table S5 Hip and knee angles for force plate trials.
(0.03 MB DOC)

Figure S1 Proximal pressure ridge in a normal human footprint.
(3.70 MB TIF)

Figure S2 Contour map of a footprint from a normal extended limb step showing lack of discernable arch (compare with Fig. 1).
(0.61 MB TIF)
