# Supplementary material for: Laetoli Footprints Preserve Earliest Direct Evidence of Human-Like Bipedal Biomechanics
Source: PLoS One. 2010 Mar 22;5(3):e9769. doi: 10.1371/journal.pone.0009769 (PMC2842428; doi:10.1371/journal.pone.0009769)
Supplement: Text S1 — Supporting Text (0.02 MB PDF) [file pone.0009769.s001.pdf]

Supporting Information

Procedures for Laetoli Footprint Scan

For the Laetoli print, a laser surface scan was taken of a high-quality cast (housed at the American Museum of Natural History, New York) of footprint G1-37. The scan was collected using a Minolta Vivid 910 (Konica Minolta Sensing, 101 Williams Drive Ramsey, NJ 07446 USA) and processed using Geomagic version 8.0 (Geomagic U.S., P.O. Box 12219, Research Triangle Park, NC, 27709 USA). Figure 1 was generated using ArcGIS after leveling the footprints.

Laetoli data collection

Values for heel and toe depths at Laetoli were taken from contour maps of the trackways [1]. The Laetoli footprints were made on a slight uphill slope (see Table S3). Therefore, we corrected the depths of the heels and toes of each print in a similar manner to the corrections made for experimentally generated prints. For each Laetoli print, we calculated the change in elevation over the length of the print, and we leveled the print so that the substrate surrounding the print was the same elevation at the heel and toe (see Table S3).

Additional Text

Kinematics

Table S4 gives the average speed and angles for all preferred speed trials through the sand trackway. Subjects walked with significantly higher degrees of flexion for the hip and knee during BKBH trials (ANOVA; $p < 0.05$ for all variables). Table S5 gives hip and knee angles for force plate trials. Hip and knee angles used during light and deep BKBH trials overlap with those used during the footprint trials.

Taxonomic ID and detecting absence of longitudinal arch in footprints

Although the Laetoli hominins clearly walked with modern human-like biomechanics, we still do not know which early hominin taxon was responsible for generating the trackways. Many researchers suggest that *Australopithecus afarensis* made these prints, based mainly on fossil evidence found nearby [2,3]. The presence of a longitudinal arch in fossils attributed to *A. afarensis* is highly debated [2,4,5]. Although the navicular has several ape-like features [4,6], indicating possible midfoot flexion, inferred insertions of ligaments on both the talus and the navicular imply there may have been a stiff mid-foot region [2,5,7]. Some suggest that the Laetoli prints also do not show evidence of an arch [8,9,10]. Meldrum [9,10] drew attention to two features of the Laetoli prints that could suggest the lack of a longitudinal arch. First, he found three prints out of 14 (21% of the prints in the trail) in the G-1 series that had a ridge proximal to the mid-foot (two other prints were described as having possible ridges; [10]). Meldrum [10] suggests these ridges develop only in the presence of mid-foot flexion, which would preclude a stiff longitudinal arch in the Laetoli hominins. However, in our sample of human footprints, we found several prints that exhibited a ridge proximal to the mid-foot (see Fig. S1). In our crouched gait sample, 25 out of 91 (27% of the total prints for BKBH trials) footprints show this ridge. In our extended limb trials, 29 out of 97 (30% of the total prints for extended limb trials) prints show this ridge. This ridge is created during push-off, as sediment is pushed towards the heel of the print.

Second, Meldrum [10] suggests that several G-1 prints lack the “narrow waisted” appearance of a human print made with a longitudinal arch. Again, we found much

variation in our sample of prints, including several that match this description (see Fig. S2).

Thus, there is ample evidence that the Laetoli prints exhibit a stiff longitudinal arch, and that variation present in Laetoli prints is also present in modern human footprints. Although the presence of a stiff arch at Laetoli supports our findings that Laetoli hominins walked with modern human-like biomechanics, the taxonomic affiliation of the Laetoli footprints must await a consensus determination of the presence of a stiff arch in *A. afarensis*. If further anatomical work demonstrates a clear longitudinal arch in *A. afarensis*, then this taxon remains the most likely candidate for attribution of the Laetoli footprints. If not, then the Laetoli prints were made by either *Kenyanthropus* (the only other hominin known from this time period; [11]), or by an as yet undiscovered hominin taxon.

Supporting Information Figure Legends

Figure S1. Proximal pressure ridge in a normal human footprint.

Figure S2. Contour map of a footprint from a normal extended limb step showing lack of discernable arch (compare with Fig. 1).

Supplementary References

1. Leakey MD, Harris JM (1987) Laetoli: A pliocene site in northern Tanzania. Oxford: Clarendon Press.
2. Stern JT, Susman RL (1983) The locomotor anatomy of *Australopithecus afarensis*. American Journal of Physical Anthropology 60: 279-317.
3. White TD (1980) Evolutionary implications of pliocene hominid footprints. Science 208: 175-176.
4. Harcourt-Smith W, Aiello L (2004) Fossils, feet and the evolution of human bipedal locomotion. Journal of Anatomy 204: 403-416.
5. Lamy P (1986) The settlement of the longitudinal plantar arch of some Africa Plio-Pleistocene hominins: a morphological study. Journal of Human Evolution 15: 31-46.
6. Sarmiento EE, Marcus LF (2000) The os navicular of humans, great apes, OH 8, Hadar, and *Oreopithecus*: Function, phylogeny, and multivariate analyses. American Museum Novitates 3288: 1-38.
7. Gebo DL (1992) Plantigrady and foot adaptation in African apes: implications for hominin origins. American Journal of Physical Anthropology 89: 29-58.
8. Bennett M, Harris J, Richmond B, Brain D, Mbua E, et al. (2009) Early hominin foot morphology based on 1.5-million-year-old footprints from Ileret, Kenya. Science 323: 1197-1201.
9. Meldrum D (2004) Midfoot flexibility, fossil footprints, and sasquatch steps: New perspectives on the evolution of bipedalism. Journal of Scientific Exploration 18: 65-79.
10. Meldrum D (2007) Renewed perspective on the Laetoli trackways: The earliest hominid footprints. In: Lucas, Spielman, Lockley, editors. Cenozoic vertebrate tracks and traces: New Mexico Museum of Natural History and Science Bulletin.
11. Leakey MG, Spoor F, Brown FH, Gathogo PN, Kiarie C, et al. (2001) New hominin genus from eastern Africa shows divers middle Pliocene lineages. Nature 410: 433-440.
