# Supplementary material for: Laetoli Footprints Preserve Earliest Direct Evidence of Human-Like Bipedal Biomechanics
Source: PLoS One. 2010 Mar 22;5(3):e9769. doi: 10.1371/journal.pone.0009769 (PMC2842428; doi:10.1371/journal.pone.0009769)
Supplement: Table S1 — Laetoli footprint data (0.03 MB DOC) [file pone.0009769.s002.doc]

Table S1. Laetoli footprint data

| Print | Heel Depth (mm) | Toe Depth (mm) | Corrected Toe Depth (mm) | Proportional Toe Depth | Grade (%) |
| --- | --- | --- | --- | --- | --- |
| G1-37 | 70.60 | 71.80 | 66.80 | 0.05 | 2.31 |
| G1-36 | 77.80 | 84.90 | 83.80 | -0.08 | 0.52 |
| G1-35 | 93.30 | 102.30 | 89.30 | 0.04 | 6.39 |
| G1-34 | 117.30 | 127.70 | 121.70 | -0.04 | 2.96 |
| G1-33 | 132.10 | 135.10 | 129.40 | 0.02 | 2.32 |
| G1-27 | 148.00 | 160.00 | 148.00 | 0.00 | 5.77 |
| G1-26 | 170.20 | 176.60 | 165.60 | 0.03 | 5.23 |
| G1-25 | 190.20 | 194.80 | 195.80 | -0.03 | -0.51 |

Note: Heel and toe depths are the deepest points for the heel and toe depressions on each prints. Note that these values are absolute elevations, so larger values are higher elevations. Corrected toe depth was calculated by leveling the print so that the heel and toe edge were at the same elevation. Proportional toe depth is the toe depth as a fraction of heel depth using the corrected toe depth values.
