# Supplementary material for: Laetoli Footprints Preserve Earliest Direct Evidence of Human-Like Bipedal Biomechanics
Source: PLoS One. 2010 Mar 22;5(3):e9769. doi: 10.1371/journal.pone.0009769 (PMC2842428; doi:10.1371/journal.pone.0009769)
Supplement: Table S2 — Subject information (0.03 MB DOC) [file pone.0009769.s003.doc]

Table S2. Subject information

| Subject | Age | Sex | Mass (kg) | Foot Length (cm) |
| --- | --- | --- | --- | --- |
| Subject 1 | 21 | F | 67.5 | 20.5 |
| Subject 2 | 20 | F | 48.7 | 23.0 |
| Subject 3 | 23 | F | 62.5 | 21.2 |
| Subject 4 | 26 | F | 73.9 | 25.8 |
| Subject 5 | 21 | M | 74.2 | 27.1 |
| Subject 6 | 20 | M | 68.0 | 25.9 |
| Subject 7 | 21 | M | 66.7 | 25.7 |
| Subject 8 | 21 | M | 63.0 | 26.0 |
