# Supplementary material for: Laetoli Footprints Preserve Earliest Direct Evidence of Human-Like Bipedal Biomechanics
Source: PLoS One. 2010 Mar 22;5(3):e9769. doi: 10.1371/journal.pone.0009769 (PMC2842428; doi:10.1371/journal.pone.0009769)
Supplement: Table S3 — Sample size information for footprint analyses (0.03 MB DOC) [file pone.0009769.s004.doc]

Table S3. Sample size information for footprint analyses

| Gait | Side | Speed | Moisture | n |
| --- | --- | --- | --- | --- |
| Normal | L | p | d | 20 |
| Normal | R | p | d | 18 |
| BKBH | L | p | d | 17 |
| BKBH | R | p | d | 19 |
| Normal | Both | p | w | 5 |
| BKBH | Both | p | w | 4 |
| Normal | L | f | d | 8 |
| Normal | R | f | d | 11 |
| BKBH | L | f | d | 9 |
| BKBH | R | f | d | 9 |

p is preferred walk; f is fast walk; d is dry (6-8% water); w is wet (10-12% water)
