# Supplementary material for: Laetoli Footprints Preserve Earliest Direct Evidence of Human-Like Bipedal Biomechanics
Source: PLoS One. 2010 Mar 22;5(3):e9769. doi: 10.1371/journal.pone.0009769 (PMC2842428; doi:10.1371/journal.pone.0009769)
Supplement: Table S4 — Speed and joint angles for trackway trials. Values are means (SEM) for all subjects. (0.03 MB DOC) [file pone.0009769.s005.doc]

Table S4. Speed and joint angles for trackway trials. Values are means (SEM) for all subjects.

| Gait | velocity (m/s) | max  hip (º) | min  hip (º) | mean  hip (º) | max  knee (º) | min knee (º) | mean knee (º) |
| --- | --- | --- | --- | --- | --- | --- | --- |
| Extended | 1.07 (0.06) | 184.52 (1.31) | 149.21 (1.84) | 168.78 (1.07) | 169.13 (1.86) | 103.22 (0.99) | 148.53 (1.38) |
| BKBH | 1.07 (0.07) | 145.10 (4.39) | 103.56 (4.17) | 122.65 (4.23) | 133.08 (4.27) | 82.46 (5.01) | 108.64 (4.84) |
