# Supplementary material for: Laetoli Footprints Preserve Earliest Direct Evidence of Human-Like Bipedal Biomechanics
Source: PLoS One. 2010 Mar 22;5(3):e9769. doi: 10.1371/journal.pone.0009769 (PMC2842428; doi:10.1371/journal.pone.0009769)
Supplement: Table S5 — Hip and knee angles for force plate trials. (0.03 MB DOC) [file pone.0009769.s006.doc]

Table S5. Hip and knee angles for force plate trials.

| Condition | Mean Hip Angle (sem) | Mean Knee Angle (sem) | N |
| --- | --- | --- | --- |
| Normal | 156.94 (0.90) | 144.78 (1.24) | 23 |
| Light BKBH | 145.39 (0.67) | 124.84 (1.39) | 23 |
| Deep BKBH | 133.34 (1.83) | 104.83 (2.93) | 18 |
