# Supplementary figures and images for: Laetoli Footprints Preserve Earliest Direct Evidence of Human-Like Bipedal Biomechanics
Source: PLoS One. 2010 Mar 22;5(3):e9769. doi: 10.1371/journal.pone.0009769 (PMC2842428; doi:10.1371/journal.pone.0009769)

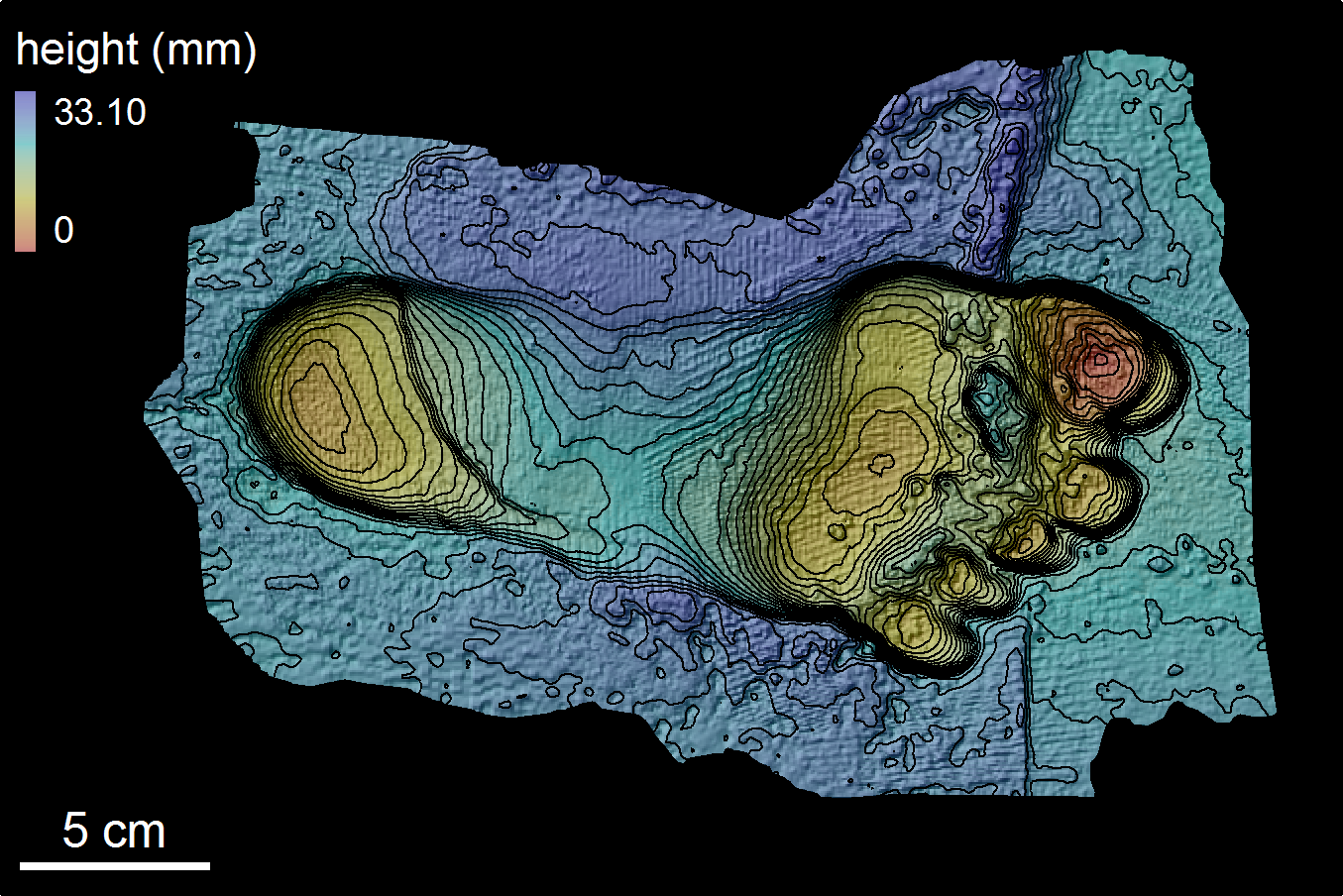

Supplement: Figure S1 — Proximal pressure ridge in a normal human footprint. (3.70 MB TIF) [file pone.0009769.s007.tif]

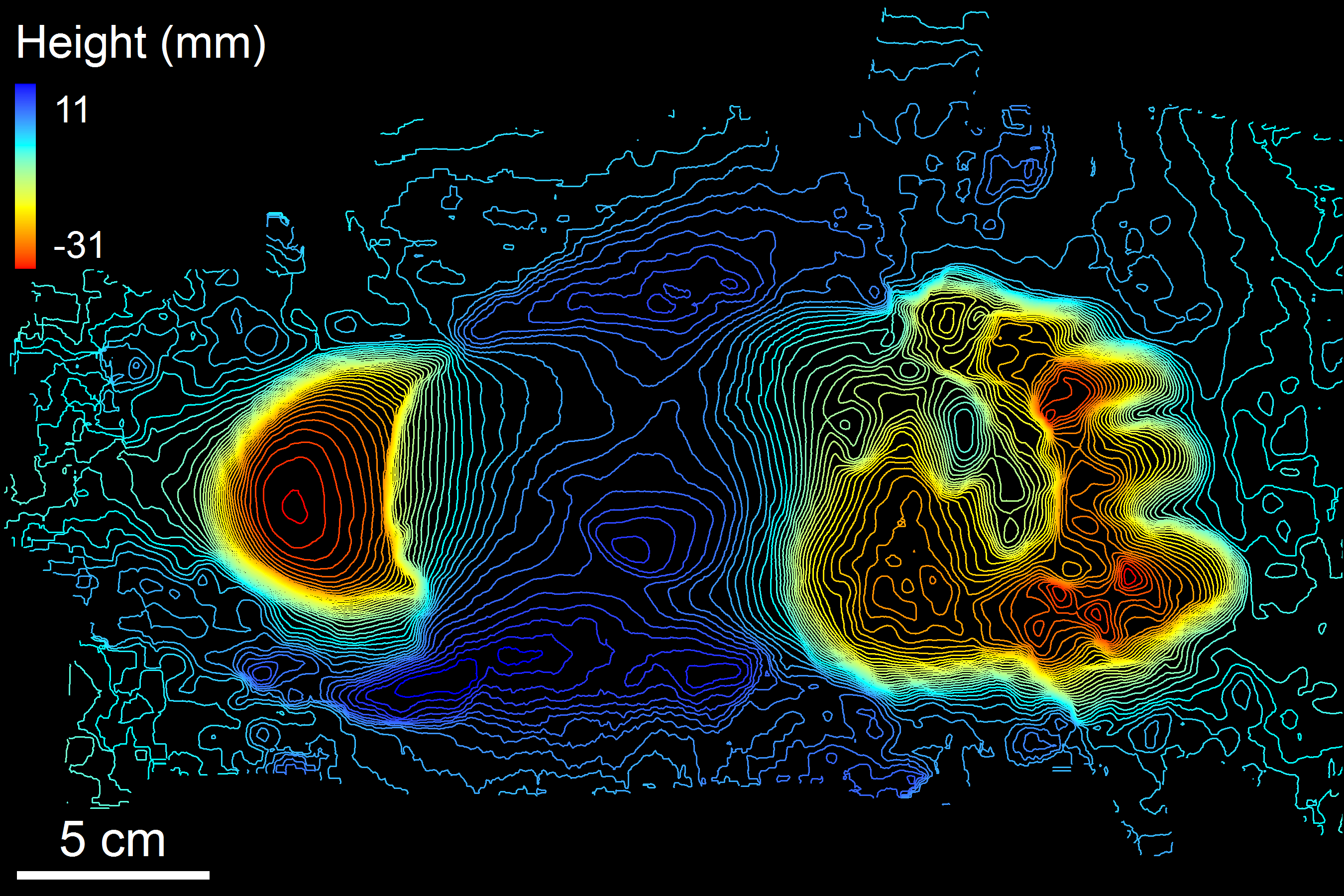

Supplement: Figure S2 — Contour map of a footprint from a normal extended limb step showing lack of discernable arch (compare with Fig. 1). (0.61 MB TIF) [file pone.0009769.s008.tif]
